# Relationships among students’ reading habits, study skills, and academic achievement in English at the secondary level

**DOI:** 10.3389/fpsyg.2023.1020269

**Published:** 2023-01-27

**Authors:** Nisar Abid, Sarfraz Aslam, Abdulelah A. Alghamdi, Tribhuwan Kumar

**Affiliations:** ^1^Department of Education, School of Social Sciences and Humanities, University of Management and Technology, Lahore, Pakistan; ^2^Faculty of Education and Humanities, UNITAR International University, Petaling Jaya, Malaysia; ^3^Faculty of Education, Umm Al-Qura University, Mecca, Saudi Arabia; ^4^Department of English Language and Literature, Prince Sattam Bin Abdulaziz University, Al-Kharj, Saudi Arabia

**Keywords:** reading, reading habits, study skills, academic achievement, secondary school students

## Abstract

**Introduction:**

Reading is an attempt to comprehend the writer’s message for personal growth and success in the relevant fields. Thus, psychologists consider it a multifaceted cognitive process of constructing meanings from texts. The present study was conducted to determine the relationships among students’ reading habits, study skills, and academic achievement in English at the secondary level in Punjab, Pakistan.

**Methods:**

The (*n* = 1614) students enrolled in the science section for the academic year 2019–2020 participated in this descriptive correlational survey, selected from 40 high schools in Lahore, Punjab, Pakistan, through a non-proportionate stratified random sampling technique. The Reading Habits Questionnaire (RHQ) and the Study Skills Scale (SSS) were used to collect data about students’ reading habits and study skills. At the same time, academic achievement was the students’ grades obtained in the ninth class in the subject of English that were determined by the Board of Intermediate and Secondary Education (BISE) Lahore in 2019. Students’ responses were analyzed through descriptive and inferential statistics.

**Results:**

The results indicated that students have competent reading habits and study skills. The correlational findings showed a strong positive relationship among reading habits, study skills, and academic achievement in English, while moderate positive relationships between reading habits and academic achievement in English. However, regression analysis results were significant, while reading habits and study skills moderately predicted academic achievement.

**Discussion:**

It is implicated that teachers should plan such assignments and tasks based on reflective thinking by considering the role of study skills in academic achievement. Moreover, teachers and school administrators could mutually create timetables for library lessons to build reading habits and study skills among learners.

## 1. Introduction

Knowledge gained through reading is vital for the cognitive, behavioral, and attitudinal development of learners (He, 2014; Baffoe and Okae-Anti, 2020; Hassan et al., 2021) because it is a person’s ability to enhance information and comprehend the words effectively (Sabbah, 2016; Al-Jarf, 2019). An individual reads for numerous reasons, i.e., knowledge development, recreation, joy, relaxation, and so on (Whitten et al., 2016). However, Erguvan (2016) and Mirza et al. (2021) directed that reading is an active part of life that is not just about pleasure when needed. However, Chotitham and Wongwanich (2014) conjectured that reading helps to develop critical and judgmental thinking abilities used to solve problems by conceptualizing context. Hence, Erdem (2015) and Pretorius and Klapwijk (2016) quantified that reading is essential to success because it starts from the commencement of school and continues throughout the lifetime.

Fischer et al. (2015), Oyewole (2017), Al-Jarf (2019) recognized that the importance of reading in learning could not be ignored because it is an emancipatory tool that releases students’ academic frustration, ignorance, and destitution. Palani (2012) distinguished that reading is an instrument used to exchange information, while reading habit is an academic activity that enables students to benefit from reading materials. Therefore, Walia and Sinha (2014) specified that reading habits require complex skills, such as perceiving a message, skimming and scanning information, and understanding the context. Thus, compelling reading depends on readers’ behaviors, known as study skills that enable them to conceptualize the new knowledge effectively (DiPerna and Elliott, 2000; Habibu and Ejembi, 2011; Gormley et al., 2018; Naqvi et al., 2018; Iheakanwa et al., 2021). While the effective study makes one narrate in their way using the stipulated meanings of the words and terms, the researchers take up for explanation and clarity (Biyik et al., 2017).

According to the available literature, students’ reading habits and study skills have been of great importance for decades; while several deficiencies were found in previous studies, thus researchers considered few of them that are related to the study context. First, the researchers mainly focused on the influence of reading habits and study skills on academic achievement separately in Western countries (Bhan and Gupta, 2010; Sabbah, 2016). A few addressed Eastern countries restricted to the university level (Demir et al., 2012; Davarci, 2013; Dilshad et al., 2013; Erguvan, 2016; Alzahrani et al., 2018; Porkaew and Fongpaiboon, 2018; Thamarasseri, 2018; Ameyaw and Anto, 2019; Ehsan and Sultana, 2020; Tonka and Bakir, 2020; Mirza et al., 2021; Nguyen Thi Thu, 2022). However, students’ reading habits and study skills may be initiated from the school level enabling the individuals to grow in competence, comfort, and understanding of the audience. At the same time, previous researchers focused on university level students’ reading habits. Second, there are methodological identities that lead to dubious findings not confirming the influence of reading habits and study skills on academic achievement (Goel, 2014; Lawrence, 2014; Quadir and Chen, 2015; Sherafat and Murthy, 2016; Ameyaw and Anto, 2018; Silverrajoo and Hassan, 2018; Balan et al., 2019; Hassan et al., 2021). In general, there is a scarcity of research aiming to determine the correlation between students’ reading habits and achievement through the role of study skills at any academic level. Finally, in Pakistan, few studies could explore reading habits as a singular variable of different groups of students (Bajwa et al., 2011; Hussain and Munshi, 2011; Rasheed, 2012). Numerous researchers only examined the relationship between reading habits and academic achievement (Bashir and Mattoo, 2012; Bibi et al., 2020; Ehsan and Sultana, 2020). Moreover, Fazal et al. (2012) only investigated the association between study skills and achievement. Thus, this research examines the relationship among students’ reading habits, study skills, and academic achievement in English as practiced at the secondary level in Lahore, Punjab, Pakistan.

Lahore is the capital of Pakistan’s Punjab province. In terms of population, this is the second largest city in Pakistan. It is located in the northeastern part of Pakistan’s Punjab province. Lahore is one of the most cosmopolitan cities in Pakistan and is home to various cultures, traditions, and customs. Specifically, it provides researchers with opportunities to contextualize perspectives in light of academic processes and ethics.

## 2. Literature review

### 2.1. Reading habits

Rosli et al. (2018) suggested that reading is an attempt to comprehend the writer’s message, while Alnahdi and Aftab (2020) stated that it is a gateway to all other information, which may lead to understanding the world outside the text. Hence, Al-Jarf (2021) and Dadzie (2008) asserted that reading is a multifaceted cognitive process of comprehending words written in a textual form that allows readers to enhance their knowledge for personal growth and academic success. Moreover, Ogeyik and Akyay (2009); Erguvan (2016), Mirza et al. (2021) stated that reading is just a method of communication between the writer and the reader. Thus, Bhan and Gupta (2010) and Baron (2017) assumed that reading is the art of decoding and interpreting messages from various written materials such as books, magazines, journals, newspapers, dictionaries, encyclopedias, pamphlets, and diaries. Hassan et al. (2021) stated that reading habits influence reading materials, activities, time duration, place of reading, and reader motivation. In this study, reading habits are considered to be the students’ reading preferences, interest in reading, attitude toward reading, and reading problems during study at the secondary level.

### 2.2. Study skills

Study skills are the readers’ inclination toward organizing, highlighting, reviewing, reciting, and using devices, flashcards, etc. to comprehend new knowledge effectively (DiPerna and Elliott, 2000; DiPerna, 2006; Rozalski, 2008; Madhavi et al., 2014; Sabbah, 2016). While reading habit is the frequency, a reader regularly reads (Winne, 2013). Moreover, study skills are the students’ intellectual practices to process new information effectively and efficiently, while reading habits are considered a psychological trait of one’s personality (Farrington et al., 2012; Pillai, 2012; Mansor et al., 2013; Shahidi et al., 2014; Ameyaw and Anto, 2018; Rosli et al., 2018). Thus, the concept of study skills is different from reading habits. This research defines study skills as secondary school students’ approaches to comprehending new knowledge.

### 2.3. Reading habits and academic achievement

Horbec (2012) and Singh (2011) determined a significant positive relationship between students’ reading habits and academic achievement. Hence, Issa et al. (2012) explored that students’ reading patterns vary and have a moderately significant influence on academic success, while Bashir and Mattoo (2012) examined that academic performance is dependent on the level of students’ study habits; thus, reading habits influence on future success, which was confirmed by Owusu-Acheaw and Larson (2014) through quantitative measures. Chotitham and Wongwanich (2014) found a moderate positive relationship between students’ study habits and achievement. However, Lawrence (2014) rejected the association between students’ academic achievement and study habits, and Goel (2014) confirmed that study habits do not influence academic performance. Therefore, Schwabe et al. (2015), Quadir and Chen (2015) concluded through a quantitative correlational study that heavy reading habits significantly impact reading efficiency; the longer the reading time, the better the results. Malik and Parveen (2016) discovered significant differences in low- and high-academic achievers’ attitudes toward study habits. They determined that high achievers are more concentrated and exhibited better study habits, good time management skills, and punctuality compared to low achievers. In the meantime, Sherafat and Murthy (2016) directed that study habits facilitate learners toward higher achievement because of their significant connections with academic achievement. Consequently, Silverrajoo and Hassan (2018) revealed divergent findings that students’ reading methods have a negative and weak relationship with academic achievement.

Ameyaw and Anto (2018) recognized the importance of reading styles in students’ learning and found that reading styles affect students’ performance. Meanwhile, Alzahrani et al. (2018) verified that students’ reading styles significantly impact their performance. Dolmaz and Kaya (2019) discovered that students’ creative writing skills are affected by their reading styles. Moreover, Balan et al. (2019) determined that students’ purpose of reading significantly affected their performance, as Annamalai and Muniandy (2013) suggested that academic performance is based on students’ reading purpose. Hence, Whitten et al. (2016) and Fatiloro et al. (2017) discovered that reading habits significantly assist students in learning and enhancing their performance. Bibi et al. (2020) examined that students’ study habits were significantly positively associated with achievement. Ehsan and Sultana (2020) predicted that reading habits significantly increase students’ performance. Moreover, Hassan et al. (2021) found a significant correlation between secondary school students’ reading habits and their reading achievement and concluded that reading habits significantly contribute to academic achievement. Thus, Nguyen Thi Thu (2022) revealed that reading habits have a significant role in the development of students writing performance.

On the other hand, by designing a correlational study, Tonka and Bakir (2020) found a negative relationship between reading anxiety and reading habits. Thus, they concluded that reading anxiety plays a role in students’ performance and reading habits. Similarly, Alnahdi and Aftab (2020) found a significant negative association between study habits and academic stress, reading habits, and academic achievement. The researchers measured all the variables through a questionnaire consisting of four scales and 43 items.

### 2.4. Study skills and academic achievement

Nouhi et al. (2009) determined that study skills have a significant positive association with academic success measured through a closed-ended questionnaire confirmed by Awang and Sinnadurai (2011) through an experimental study. Meanwhile, Hassanbeigi et al. (2011) and Sabbah (2016) verified that study skills are critical for academic success because they positively correlate with academic achievement found through a descriptive correlational survey using a study skills scale. Hence, Fazal et al. (2012) suggested that higher academic achievers use a wide range of study skills than low achievers, while there was a weak correlation between study skills and academic success. Furthermore, Demir et al. (2012) revealed through an experimental study that students’ study skills had a considerable influence on performance which was also confirmed by Wernersbach et al. (2014) from an experimental study. In both of the studies, researchers measured study skills through closed-ended items. Moreover, they also discovered that study skills significantly impact students’ academic self-efficacy. Nonetheless, Tahamtani et al. (2017) and Naqvi et al. (2018) revealed a weak negative link between achievement and study habits through quantitative measures that were rejected by Gormley et al. (2018), who found a significant positive impact of study skills on achievement.

Several gaps were found in already conducted studies; first, the researchers mainly focused on the influence of reading habits and study skills on academic achievement separately in Western countries, while few addressed this phenomenon in Eastern countries. However, the investigation was restricted to university level students. Second, methodological identities lead to dubious findings not confirming this phenomenon. Finally, in Pakistan, few studies could explore reading habits as a singular variable of different groups of students. At the same time, some researchers only examined the relationship between reading habits and academic achievement. Thus, this study aimed to develop our understanding of the relationship between students’ reading habits, study skills, and academic achievement in English.

### 2.5. Summary

Reading is an attempt to comprehend the writer’s message for personal growth and success. Thus, psychologists consider it a multifaceted cognitive process of constructing meaning from texts. Bhan and Gupta (2010) stated that reading is the art of decoding and interpreting messages from the content of the written material that is often carried out in magazines, journals, newspapers, books, dictionaries, encyclopedias, pamphlets, diaries, and so on. While reading habits are the degree to which a reader engages in reading while studying skills to gain knowledge. Reading habits assist students in learning more, whereas study skills encourage them to understand new information effectively. Both reading habits and study skills influence students’ academic performance.

Based on literature insights, the following hypotheses are formulated:

*Hypothesis (H_1_): A significant relationship exists between students’ reading habits and their academic achievement in English language comprehension*.

*Hypothesis (H_2_): A significant relationship exists between stqudents’ study skills and their academic achievement in English language comprehension.*

## 3. Methods

### 3.1. Design

A research design is comprised of numerous elements (i.e., research paradigm, research approach, research design, and data collection method that provide guidelines for carrying out the study (Creswell and Clark, 2017; Myers, 2019), while a correlational research design is used to determine the relationship between two or more than two variables (Cohen et al., 2018). Thus, a correlational research design of a quantitative approach (positivism paradigm) was used. At the same time, a cross-sectional survey method was applied to collect data about studied variables (i.e., reading habits, study skills, and academic achievement in English).

### 3.2. Sample

The sample comprised 10th-grade students enrolled in district Lahore’s public sector high schools for the academic year 2019–2020. The inclusion criteria were those students who enrolled in the science section only. During the data collection, the total number of active students in both sections (i.e., science and arts) of 10th grade was 36,847 enrolled at 334 high schools in district Lahore (Government of Punjab [GOP], 2019). While in the science section, the active students were 17,028, considered an accessible population of this study. A total of 1,800 (900 boys and 900 girls) were selected from 40 high schools through a non-proportionate random sampling technique that was 10.57% of the accessible population, which shows the sample was normally distributed. Out of 1,800 selected students, 1,619 participated as respondents because 181 students had not passed the subject English in the ninth-grade annual examination conducted by the Board of Intermediate and Secondary Education (BISE) Lahore. Three students declined to participate in this survey, while two could not complete the questionnaires. Therefore, the final sample consisted of *n* = 1,614 secondary school students.

### 3.3. Instruments

Two instruments were used, i.e., the Reading Habits Questionnaire (RHQ) and Study Skills Scale (SSS), to collect data about students’ reading habits and study skills.

Reading Habits Questionnaire (RHQ): The researchers developed a paper and pencil student self-report RHQ based on Ajzen’s (1991) Theory of Planned Behavior (TPB) and the social-cognitive theory of self-regulated learning strategies (Pintrich et al., 1993; Duncan and McKeachie, 2005; Duncan et al., 2007). Ajzen’s (1991) TPB suggests that socio-psychological characteristics of a person’s behavior, such as reading, influence reader proximal behaviors (Stokmans, 1999; Miesen, 2003; Van Schooten et al., 2004), while the social-cognitive theory of self-regulating learning strategies suggested that students’ reading habits are meta-cognitively and behaviorally active in a student’s learning process to achieve goals (Eccles and Wigfield, 2002). The classical test theory model was utilized to develop RHQ, which initially consisted of 44 closed-ended items. Each item was constructed on a 5-point Likert-type agreement scale ranging from 1 (strongly disagree) to 5 (strongly agree), which means developing level reading habits to advance level reading habits. However, content validity was ensured by five education and assessment experts to validate the content coverage, language appropriateness, and usability of RHQ at the secondary level. Moreover, a pilot study was conducted on 250 students selected purposively from the target population to confirm unidimensionality among items and scales through exploratory factor analyses (EFAs) using Statistical Package for Social Sciences (SPSS) Version 23 software. Four subscales of RHQ (i.e., preferences for reading, interest in reading, attitude toward reading, and reading problems) were constructed during EFA. In contrast, nine items (two to three from each subscale) were deleted because their factor loading values (λ) were less than 0.5. In an analysis of items, reliability was also determined through Cronbach’s alpha (α = 0.821) value which was statistically acceptable. Psychometric evidence shows that RHQ was reliable for determining students’ reading habits. Improved RHQ consisted of 35 items based on four subscales, i.e., preferences of reading (10 items), interest in reading (nine items), attitude toward reading (nine items), and reading problems (seven items).

Study Skills Scale (SSS): The researchers adopted the SSS from Academic Competence and Evaluation Scale, developed by DiPerna and Elliott in 2000. The validity, as well as reliability of SSS, was confirmed by numerous researchers (Kettler et al., 2014; Strunk, 2014; Anthony and DiPerna, 2018) and concluded that SSS is a standardized scale to measure study skills. The SSS consisted of 11 items that were also constructed on a 5-point Likert-type frequency scale, ranging from 1 (never) to 5 (almost always) which means developing level to advance level skills (DiPerna and Elliott, 2000). The SSS was also administered to 250 students to ensure reliability through Cronbach’s alpha tests and found a value of α = 0.874 that was suitable to measure study skills in the local context (Pakistan).

Academic Achievement: Students’ marks obtained in ninth grade in the subject of English were asked them that determined by the Board of Intermediate and Secondary Education (BISE) Lahore in 2019. Their obtained scores in the subject of English were considered an academic achievement of students.

### 3.4. Data collection and analysis

After getting consent from the district education administration officer, the researchers personally gained permission from the selected schools’ principals and class teachers for data collection. All the selected students were informed in their classes about the study purpose and given the right to withdraw from the study at any time before data analyses. RHQ and SSS administration occurred over 8 weeks during mid of the September to mid of November 2019 academic year. Before administering the instruments, participants were informed about the confidentiality procedures. Moreover, the researchers encouraged them to respond honestly and told them to write about their obtained marks in ninth grade in English. After collecting the questionnaires, the researchers quickly scanned the participants’ responses about missing answers, and the students were asked to complete the responses in the questionnaire. A total of 1,614 participants provided valuable responses about their reading habits and study skills. The response rate was 89.6%, acceptable in social sciences research for quantitative data. Students’ responses about reading habits and study skills were analyzed by applying descriptive statistics (i.e., mean, standard deviation, skewness, and kurtosis) and inferential statistics (i.e., Pearson *r* test and regression test) through SPSS version 25 software.

## 4. Results

### 4.1. Descriptive statistics on students’ reading habits and study skills

The Kolmogorov–Smirnov test was used to check the normality of data. At the same time, the skewness and kurtosis values indicated that data were normally distributed because skewness and kurtosis were between –2 and + 2, which was suitable for parametric statistics (George, 2011; Albers, 2017; Mishra et al., 2019). Table 1 indicates that students give more preferences to reading than their attitude toward reading, interest in reading, and reading problems as *M* = 4.13, *SD* = 0.486; *M* = 3.91, *SD* = 0.616; *M* = 3.68, *SD* = 0.676; and *M* = 3.57, *SD* = 0.813, respectively. Students also thought they were facing reading problems because English was not their native language as *M* = 3.68; *SD* = 0.813. Overall, results revealed that students have permissive and desired reading habits and study skills as *M* = 3.88, *SD* = 0.455 and *M* = 3.85, *SD* = 0.602.

**TABLE 1:** Descriptive statistics of reading habits and study skills.

Scales	*Mean*	*SD*	Skewness	Kurtosis
Preferences of reading	4.13	0.486	−0.632	0.623
Interest in reading	3.68	0.676	−0.871	0.546
Attitude toward reading	3.91	0.616	−1.027	1.162
Reading problems	3.68	0.813	−1.024	1.219
Reading habits-total score	3.88	0.455	−0.987	1.047
Study skills	3.85	0.602	−0.893	1.016

*N* = 1614.

### 4.2. Inferential statistics on students’ reading habits, study skills, and academic achievement in English

The Pearson product–moment correlation analysis was applied to test null hypotheses, i.e., whether there are significant relationships among students’ reading habits, study skills, and academic achievement in English.

### 4.3. Hypotheses testing

Refer to Table 2 for the intercorrelation of variables among reading habits subscales, overall reading habits, and study skills; there were moderate-to-high positive correlations among variables. For reading habits, study skills, and academic achievement in English, students’ reading habits were significantly and positively correlated as *r* = 0.314–0.721. A value of *r* = (±) 0.3–0.7 exhibits a moderate-to-high correlation between variables (Akoglu, 2018; Schober et al., 2018). Results also reveal higher positive correlations between reading habits and study skills while moderate positive correlations between reading habits and academic achievement in English as *r* = 0.848 and 0.584, respectively. Moreover, there was a higher positive correlation between study skills and academic achievement in English as *r* = 0.721. Thus, it is revealed that students’ reading habits and study skills are positively associated with academic achievement in English.

**TABLE 2:** Intercorrelations matrix and relationships among students’ reading habits, study skills, and academic achievement in English.

	PR	IR	AR	RP	RHT	SS	AAE
PR	–						
IR	0.703**	−					
AR	0.761**	0.644**	−				
RP	0.781**	0.752**	0.734**	−			
RHT	0.862**	0.833**	0.819**	0.808**	–		
SS	0.883**	0.875**	0.867**	0.859**	0.848**	–	
AAE	0.591**	0.713**	0.418**	0.314**	0.584**	0.721**	–

PR, preferences of reading; IR, interest in reading; AR, attitude toward reading; RP, reading problems; RHT, reading habits total score; SS, study skills; AAE, academic achievement in English; *N*, 1614; and **correlation is significant at the 0.01 level (two-tailed).

Since the hypotheses “there is a significant relationship between students’ reading habits and academic achievement in English, and there is a significant relationship between students’ study skills and academic achievement in English” were accepted because moderate-to-strong positive relationships were found among students’ reading habits, study skills, and academic achievement in English.

A regression analysis was conducted to explore whether students’ reading habits and study skills predict academic achievement in English. Students’ reading habits and study skills served as independent variables, while students’ academic achievement in English served as dependent variables. The regression analysis results were significant. The unique individual predictor for students’ academic achievement in English interested in reading and attitude toward reading. These two sub-factors of reading habits significantly predicted 42 and 43% of the variance, respectively.

In contrast, the numeric regression does not considerably reveal the remaining two sub-factors (preferences of reading and reading problems). However, students’ reading habits accounted for 44% of the variance, and study skills accounted for 48% of the variance. Refer to Table 3 for unstandardized betas, standard errors, standardized betas, and adjusted *R*^2^. The independent variables in these analyses are moderately correlated and predict academic achievement because the variance inflation factor (VIF) estimation was below 5.0 in regression.

**TABLE 3:** Summary of regression analyses, with 95% confidence intervals, of students’ reading habits and study skills predicting academic achievement in English.

Dependent variable	Independent variables	*B*	Lower	Upper	*SE B*	β	Adjusted *R*^2^	*p*
AAE	PR	0.015	−0.13	0.042	0.01	0.16	0.05	0.060
	IR	0.017	−0.010	0.039	0.01	0.18	0.42**	0.020
	AR	0.016	−0.014	0.044	0.01	0.19	0.43**	0.035
	RP	0.018	−0.16	0.038	0.01	0.25	0.05	0.087
	RHT	0.013	−0.010	0.038	0.01	0.21	0.44**	0.002
	SS	0.016	−0.013	0.043	0.01	0.27	0.48**	0.001

PR, preferences of reading; IR, interest in reading; AR, attitude toward reading; RP, reading problems; RHT, reading habits total score; SS, study skills; AAE, academic achievement in English; and **p < 0.001.

## 5. Discussion

Reading habit is a crucial aspect of creating a literate society because it helps to shape personality, develop creative and critical thinking abilities, and enhance knowledge (Palani, 2012; Mansor et al., 2013; Fischer et al., 2015; Bano et al., 2018; Rosli et al., 2018; Al-Jarf, 2019; Wu et al., 2019; Hassan et al., 2021). At the same time, study skills are the readers’ strategies to process new information effectively (Kuterbach, 2012; Anthony and DiPerna, 2018; Abid et al., 2021). Both reading habits and study skills are interdependent and influence students’ academic performance as well as future success (Demir et al., 2012; Wernersbach et al., 2014; Tahamtani et al., 2017; Alzahrani et al., 2018; Ameyaw and Anto, 2018; Gormley et al., 2018; Balan et al., 2019; Dolmaz and Kaya, 2019; Ehsan and Sultana, 2020). Therefore, this study is designed to examine relationships among students’ reading habits, study skills, and academic achievement at the secondary level in Lahore, Pakistan. Lahore is one of the cosmopolitan cities of Pakistan and a hub of many cultures, traditions, and customs. Regarding the academic processes and ethics, it provides opportunities for researchers to contextualize the perspectives accordingly. Reading habits have been and are still being taught in schools, colleges, and universities through model reading by teachers, parents, or elders of the families. In addition, the reading and recitation of fold tales and poems get to gathers like at Pak Tea House, Lawrence Garden, Quaid-e-Azam Library, and so on, while formal schools books, extra reading exercises, and reading and writing competitions at the school level are prepared through a variety of book reading within the context of the particular objective. The results of normality tests indicated that the data were normality distributed and suitable to apply parametric statistics. The descriptive findings also showed that students have more preferences for reading than their attitude toward reading, interest in reading, and reading challenges. These results support the finding of numerous researchers (e.g., Pehlivan et al., 2010; Mansor et al., 2013; Owusu-Acheaw and Larson, 2014; Haliru et al., 2015; Erguvan, 2016; Krashen, 2016; Kulatunga, 2016; Loan and Shah, 2017; Ameyaw and Anto, 2018; Porkaew and Fongpaiboon, 2018; Mirza et al., 2021) who found that students give more preference to read academic content from textbooks and other reading materials (i.e., newspaper, storybooks, poetry, novel, magazines, cartoons, comics, sports, etc.). Rasheed (2012) determined that reading habits play a substantial essential role in developing positive attitudes toward reading. However, Maiyo and Siahi (2015) revealed that higher achievers had better reading habits than low achievers. Students prefer reading online because they can easily read content from the internet material in this technological age, so they prefer reading online (Dollah et al., 2017). Thus, Molotja and Themane (2018) found that students’ reading habits may enhance through global reading strategies and problem-solving strategies. Moreover, it is found that students have competence in reading habits and study skills. These results are also in line with the findings of previous studies, e.g., Dadzie (2008), Ogeyik and Akyay (2009), Bhan and Gupta (2010), and Issa et al. (2012), and Sabbah (2016) revealed that the majority of the students read books to pass the exams that why they have good reading habits. Furthermore, it is determined that students also possess the competence level of study skills that confirmed the study conducted by numerous researchers (i.e., DiPerna, 2004, 2006; Rozalski, 2008; Kuterbach, 2012; DuPaul and Stoner, 2014; Anthony and DiPerna, 2018; Abid et al., 2021).

Furthermore, researchers also concluded from correlational results that there were moderate-to-significant positive correlations among reading habits, study skills, and academic achievement in English. In the literature review, it is seen that these findings are consistent with the results of Singh (2011), Horbec (2012), Issa et al. (2012), Sabbah (2016), Ameyaw and Anto (2018), Hassan et al. (2021). They found a positive relationship between reading habits and academic achievement, while reading habits influence students’ academic performance. Moreover, some researchers determined a moderate relationship between reading habits and academic success (e.g., Chotitham and Wongwanich, 2014; Kutay, 2014; Owusu-Acheaw and Larson, 2014; Alzahrani et al., 2018; Adigun et al., 2021; Nguyen Thi Thu, 2022). Sherafat and Murthy (2016) directed that study habits facilitate learners toward higher achievement because of their significant connections with academic achievement, that confirmed by Bibi et al. (2020). In contrast, few researchers found different results due to participants’ different selection procedures and contextual differences (i.e., purposive sample method, content, reading material, culture, etc.). For example, Lawrence (2014); Goel (2014), Alnahdi and Aftab (2020) revealed no significant correlation between students’ academic achievement and study habits. At the same time, Silverrajoo and Hassan (2018) found that students’ reading styles have a negative, weak relationship with academic achievement. Findings regarding study skills: e.g., Nouhi et al. (2009), Awang and Sinnadurai (2011), Hassanbeigi et al. (2011), Maiyo and Siahi (2015), Gormley et al. (2018) revealed a significant positive connection between study skills and academic success, whereas Demir et al. (2012) and Wernersbach et al. (2014) found study skills have a considerable influence on performance that support the present study findings. However, few researchers found a negative correlation between study skills and academic performance (Fazal et al., 2012; Tahamtani et al., 2017; Naqvi et al., 2018). Furthermore, researchers determined in this study students’ reading habits have positive correlations with study skills. Thus, reading habits and study skills directly correlate with their academic achievement in English. In addition, it is also revealed that reading habits and study skills moderately predict students’ academic achievement. Annamalai and Muniandy (2013) suggested that academic performance is based on students’ reading habits. Whitten et al. (2016) and Fatiloro et al. (2017) revealed that reading habits significantly help students learn more to enhance their academic performance. Ehsan and Sultana (2020) predicted that reading habits significantly improve students’ academic performance.

## 6. Conclusion

Reading habits and study skills differ in conceptual understanding. Reading habits are the degree to which readers regularly read, whereas study skills are the ability to comprehend new information effectively. Both reading and study habits influence students’ academic performance. It is concluded that the collected data were normally distributed. The descriptive findings about reading habit sub-constructs indicated that students give more preferences to reading than their attitude toward reading, interest in reading, and reading problems. Simultaneously, they have competent reading habits and study skills. Furthermore, it is found that there are moderate-to-strong positive correlations among reading habits, study skills, and academic achievement in English. Thus, it is concluded that reading habits and study skills directly correlate with academic achievement in English. In addition, it is also revealed that reading habits and study skills moderately predict students’ academic achievement.

## 7. Implications for practice

It is determined that students prefer reading to their attitude toward reading, interest in reading, and challenges and have competent reading habits and study skills. Thus, it is suggested that teachers plan such assignments and tasks based on reflective thinking (Aslam et al., 2021), so students have to visit the school library to read more academic material to accomplish assigned tasks through extensive reading. Students’ reading habits and study skills have a moderate-to-strong connection with their academic achievement in English. So school administrations design a timetable by consulting with teachers, allowing students to spend at least an hour in the library regularly. In contrast, the library should have up-to-date reading material, exciting storybooks, and stock which attract students. In addition, parents can also engage their children in constant reading at home by providing related textbook materials and allowing them to watch educational television programs to gain the essence of reading habits and study skills.

## 8. Limitations and implications for future research

There are several limitations to this study. First, this study was conducted on secondary school students by selecting a sample from the Lahore district of Punjab, Pakistan. Therefore, future studies may include participants from other districts of Punjab and other provinces of Pakistan to increase the generalizability of results. Second, longitudinal studies are needed to explore the change in students reading habits and study skills over time. To enhance reading habits and study skills, interventional studies may build lifelong reading habits and study skills among learners to make a scholarly society. Last but not least, future researchers may explore parent’s role in developing their children’s reading habits and study skills by selecting participants from diverse populations. Cultural factors would affect students’ reading habits; thus, Pakistan’s unique culture should be considered a potential theoretical explanation in future.

## Data availability statement

The raw data supporting the conclusions of this article will be made available by the authors, without undue reservation.

## Ethics statement

Ethical review and approval was not required for the study on human participants in accordance with the local legislation and institutional requirements. Written informed consent to participate in this study was provided by the participants or their legal guardian/next of kin.

## Author contributions

NA presented the main idea and wrote the first draft of the manuscript. SA contributed to conducting the methodology. SA, AA, and TK were involved with the revisions and proofreading. All authors contributed to the article revisions and approved the submitted version.

## References

[B1] Abid N. Ali R. Akhter M. (2021). Exploring gender-based difference towards academic enablers scales among secondary school students of Pakistan. *Psychol. Sch.* 58 1380–1398. 10.1002/pits.22538

[B2] Adigun I. O. Oyewusi F. O. Aramide K. A. (2021). The impact of Covid-19 pandemic lockdown on reading engagement of selected secondary school students in Nigeria. *Interdiscip. J. Educ. Res.* 3 45–55. 10.51986/ijer-2021.vol3.01.05

[B3] Ajzen I. (1991). The theory of planned behavior. *Organ. Behav. Hum. Decis. Process.* 50 179–211. 10.1016/0749-5978(91)90020-T

[B4] Akoglu H. (2018). User’s guide to correlation coefficients. *Turkish J. Emerg. Med.* 18 91–93. 10.1016/j.tjem.2018.08.001 30191186 PMC6107969

[B5] Albers M. J. (2017). *Introduction to quantitative data analysis in the behavioral and social sciences.* Hoboken, NJ: John Wiley & Sons, 10.1002/9781119290384

[B6] Al-Jarf R. (2021). Collaborative mobile ebook reading for struggling EFL college readers. *IOSR J. Res. Method Educ.* 11 32–42.

[B7] Al-Jarf R. (2019). Quality in teaching reading to high school students. *Eurasian Arabic Stud.* 5 36–62.

[B8] Alnahdi A. S. Aftab M. (2020). Academic stress, study habits and academic achievement among university students in Jeddah[Special Issue]. *Int. J. Psychosoc. Rehabil.* 24 97–104. 10.37200/IJPR/V24SP1/PR201138

[B9] Alzahrani S. S. Soo Park Y. Tekian A. (2018). Study habits and academic achievement among medical students: A comparison between male and female subjects. *Med. Teach.* 40 1–9. 10.1080/0142159X.2018.1464650 29909709

[B10] Ameyaw S. K. Anto S. K. (2018). Read or perish: Reading habits among students and its effect on academic performance: A case study of eastbank senior high school-accra. *Libr. Philos. Pract.* 1–23. Available online at: https://digitalcommons.unl.edu/libphilprac/1748

[B11] Ameyaw S. Anto S. K. (2019). Gender variation in reading habits in schools in Moland: A case study of Asantekwaa SDA Junior High School. *Eur. J. Educ. Stud.* 6 250–264. 10.5281/zenodo.3473923

[B12] Annamalai S. Muniandy B. (2013). Reading habit and attitude among Malaysian polytechnic students. *Int. Online J. Educ. Sci.* 5 32–41.

[B13] Anthony C. J. DiPerna J. C. (2018). Piloting a short form of the academic competence evaluation scales. *Sch. Ment. Health* 10 314–321. 10.1007/s12310-018-9254-7

[B14] Aslam S. Hali A. U. Zhang B. H. Saleem A. (2021). *The Teacher Education Program’s Impact on Preservice Teachers’ Reflective Thinking in Pakistan.* Thousand Oaks, CA: Sage Open, 10.1177/21582440211055724

[B15] Awang M. Sinnadurai S. K. (2011). A study on the development of strategic tools in study orientation skills towards achieving academic excellence. *J. Lang. Teach. Res.* 2 60–67. 10.4304/jltr.2.1.60-67

[B16] Baffoe G. A. Okae-Anti A. (2020). Reading habits of selected communication educators in Ghana. *J. Educ. Pract.* 11 45–51.

[B17] Bajwa N. Gujjar A. Shaheen G. Ramzan M. (2011). A comparative study of the study habits of the students from formal and non-formal systems of education in Pakistan. *Int. J. Bus. Soc. Sci.* 2 175–186.

[B18] Balan S. Katenga J. E. Simon A. (2019). Reading habits and their influence on academic achievement among students at Asia pacific international university. *Abstr. Proc. Int. Scholars Conf.* 7 1490–1516. 10.35974/isc.v7i1.928

[B19] Bano J. Jabeen Z. Qutoshi S. B. (2018). Perceptions of teachers about the role of parents in developing reading habits of children to improve their academic performance in schools. *J. Educ. Educ. Dev.* 5 42–59. 10.22555/joeed.v5i1.1445

[B20] Baron N. S. (2017). Reading in a digital age. *Phi Delta Kappan* 99 15–20. 10.1177/003172171773418

[B21] Bashir I. Mattoo N. H. (2012). A study on study habits and academic performance among adolescents (14-19) years. *Int. J. Soc. Sci. Tomorrow* 1 1–5.

[B22] Bhan K. S. Gupta R. (2010). Study habits and academic achievement among the students belonging to scheduled caste and non-scheduled caste group. *J. Appl. Res. Educ.* 15 1–9.

[B23] Bibi A. Naseer N. Habib Z. (2020). Study habits of students and academic achievement: A correlational study. *Glob. Educ. Stud. Rev.* 5 114–122. 10.31703/gesr.2020(V-III).12

[B24] Biyik M. A. Erdogan T. Yildiz M. (2017). The examining reading motivation of primary students in the terms of some variables. *Int. J. Prog. Educ.* 13 31–49.

[B25] Chotitham S. Wongwanich S. (2014). The reading attitude measurement for enhancing elementary school students’ achievement. *Procedia Soc. Behav. Sci.* 116 3213–3217. 10.1016/j.sbspro.2014.01.737

[B26] Cohen L. Manion L. Morrison K. (2018). *Research methods in education*, 8th Edn. Milton Park: Routledge.

[B27] Creswell J. W. Clark V. L. P. (2017). *Designing and conducting mixed methods research.* Thousand Oaks, CA: Sage.

[B28] Dadzie P. S. (2008). Reading for education: The roles of libraries. *Ghana Libr. J.* 20 1–14. 10.4314/glj.v20i1.33978

[B29] Davarci N. (2013). *An investigation on the evaluation of the relationship between the reading habits of 8th grade elementary school students and their habits of computer-internet usage* [Unpublished master thesis]. Nigeria: University of Nigeria.

[B30] Demir S. Kilinc M. Dogan A. (2012). The effect of curriculum for developing efficient studying skills on academic achievements and studying skills of learners. *Int. Electron. J. Elem. Educ.* 4 427–440.

[B31] Dilshad M. Adnan A. Akram A. (2013). Gender differences in reading habits of university students: An evidence from Pakistan. *Pak. J. Soc. Sci. (PJSS)* 33 311–320.

[B32] DiPerna J. C. (2004). Structural and concurrent validity evidence for the academic competence evaluation scales-college edition. *J. Coll. Couns.* 7 64–72. 10.1002/j.2161-1882.2004.tb00260.x

[B33] DiPerna J. C. (2006). Academic enablers and student achievement: Implications for assessment and intervention services in the schools. *Psychol. Sch.* 43 7–17. 10.1002/pits.20125

[B34] DiPerna J. C. Elliott S. N. (2000). Academic competence evaluation scales. *Psychol. Corp.* 10.1037/t14965-000

[B35] Dollah W. A. K. W. Fakeh S. S. K. W. Kamal Rafedzi E. R. Ibrahim A. Rahim H. Masron M. Z. A. (2017). Inculcating reading habits among secondary school students. *J. Sci. Eng. Res.* 4 407–421.

[B36] Dolmaz M. Kaya E. (2019). The effect of 7th grade students’ reading habits and their academic achievement in social studies and Turkish courses on their creative writing skills. *Int. Online J. Educ. Sci.* 11 168–183. 10.15345/iojes.2019.01.012

[B37] Duncan G. J. Dowsett C. J. Claessens A. Magnuson K. Huston A. C. Klebanov P. (2007). School readiness and later achievement. *Dev. Psychol.* 43 1428–1446. 10.1037/0012-1649.43.6.1428 18020822

[B38] Duncan T. G. McKeachie W. J. (2005). The making of the motivated strategies for learning questionnaire. *Educ. Psychol.* 40 117–128. 10.1207/s15326985ep4002_6 26627889

[B39] DuPaul G. J. Stoner G. (2014). *ADHD in the schools: Assessment and intervention strategies*, 3rd Edn. New York, NY: Guilford Press.

[B40] Eccles J. S. Wigfield A. (2002). Motivational beliefs, values, and goals. *Ann. Rev. Psychol.* 53 109–132. 10.1146/annurev.psych.53.100901.135153 11752481

[B41] Ehsan T. Sultana N. (2020). Predicting the role of study habits in academic achievement: A study of university students in Punjab. *Pak. J. Educ.* 37 95–112. 10.30971/pje.v37i1.1410

[B42] Erdem A. (2015). A research on reading habits of university students: Sample of Ankara University and Erciyes University. *Procedia Soc. Behav. Sci.* 174 3983–3990. 10.1016/j.sbspro.2015.01.1145

[B43] Erguvan D. (2016). ’Students’ attitudes towards extensive and intensive reading and ‘instructors’ motivational strategies. *Arab World Eng. J. (AWEJ)* 7 136–150.

[B44] Farrington C. A. Roderick M. Allensworth E. Nagaoka J. Keyes T. S. Johnson D. W. (2012). *Teaching adolescents to become learners. The role of noncognitive factors in shaping school performance: A critical literature review.* Chicago, IL: University of Chicago Consortium on Chicago School Research.

[B45] Fatiloro O. F. Adesola O. A. Hameed B. A. Adewumi O. M. (2017). A survey on the reading habits among colleges of education students in the information age. *J. Educ. Pract.* 8 106–110.

[B46] Fazal S. Hussain S. Majoka M. I. Masood S. (2012). The role of study skills in academic achievement of students: A closer focus on gender. *Pak. J. Psychol. Res.* 27 37–51.

[B47] Fischer L. Hilton J. Robinson T. J. Wiley D. A. (2015). A multi-institutional study of the impact of open textbook adoption on the learning outcomes of post-secondary students. *J. Comput. High. Educ.* 27 159–172. 10.1007/s12528-015-9101-x 32269452 PMC7115070

[B48] George D. (2011). *SPSS for Windows step by step: A simple study guide and reference, 17.0 update*, 10th Edn. London: Pearson Education.

[B49] Goel U. (2014). Comparative study of study habits in relation to academic achievement of senior secondary school students. *Gyanodaya J. Prog. Educ.* 7 18–25. 10.5958/2229-4422.2014.00004.8

[B50] Gormley M. J. Pinho T. Pollack B. Puzino K. Franklin M. K. Busch C. (2018). Impact of study skills and parent education on first-year GPA among college students with and without ADHD: A moderated mediation model. *J. Atten. Disord.* 22 334–348. 10.1177/1087054715594422 26187415 PMC4715995

[B51] Government of Punjab [GOP] (2019). *Census of school education department.* Lahore: Government of Punjab.

[B52] Habibu I. Ejembi S. (2011). The role of schools and public libraries in promoting reading habit among children and adolescents in Nigeria. *Inf. Knowl. Manag.* 1 33-40.

[B53] Haliru R. A. Abdulkarim M. Mohammed A. D. Dangani B. U. (2015). An assessment of reading habit among secondary school students in Kaduna metropolis. *J. Hum. Soc. Sci.* 20 12–17.

[B54] Hassan I. Latiff Azmi M. N. Muhamad S. N. Abdullah A. T. H. (2021). Reading habits and their correlation with reading achievement among ESL learners in selected Malaysian secondary schools. *Arab World Eng. J. (AWEJ)* 12 385–399. 10.24093/awej/vol12no3.27

[B55] Hassanbeigi A. Askari J. Nakhjavani M. Shirkhoda S. Barzegar K. Mozayyan M. R. (2011). The relationship between study skills and academic performance of university students. *Procedia Soc. Behav. Sci.* 30 1416–1424. 10.1016/j.sbspro.2011.10.276

[B56] He M. (2014). “Extensive reading and students’ academic achievement: A case study,” in *Exploring EFL fluency in Asia*, eds Muller T. Adamson J. Brown P. S. Herder S. (Berlin: Springer), 231–243. 10.1057/9781137449405_14

[B57] Horbec D. (2012). The link between reading and academic success. *Eng. Aust.* 47 58–67.

[B58] Hussain I. Munshi P. (2011). Identifying reading preferences of secondary school students. *Creat. Educ.* 2 429–434. 10.4236/ce.2011.25062

[B59] Iheakanwa J. U. Obro S. Akpochafo W. P. (2021). Reading ability, study habits and students’ academic performance in social studies. *Libr. Philos. Pract.* 1–21. Available online at: https://digitalcommons.unl.edu/libphilprac/5675

[B60] Issa A. O. Aliyu M. B. Akangbe R. B. Adedeji A. F. (2012). Reading interests and habits of the federal polytechnic, OFFA, students. *Int. J. Learn. Dev.* 2 470–486. 10.5296/ijld.v2i1.1470

[B61] Kettler R. J. Elliott S. N. DiPerna J. C. Bolt D. M. Reiser D. Resurreccion L. (2014). Student and teacher ratings of academic competence: An examination of cross-informant agreement. *J. Appl. Sch. Psychol.* 30 338–354. 10.1080/15377903.2014.950442

[B62] Krashen S. (2016). “Compelling reading and problem-solving: The easy way (and the only way) to high levels of language, literacy, and life competence,” in *Proceedings of the epoch making in english language teaching and learning twenty-fifth international symposium on english teaching, english teachers*, Taipei, 115–125.

[B63] Kulatunga R. K. (2016). *A study on understanding the reading habits and library usage of under graduate students (2007/2008 batch) of Uva Wellassa University of Sri Lanka.* [Unpublished doctoral dissertation]. Badulla: Uva Wellassa University of Sri Lanka.

[B64] Kutay V. (2014). *A survey of the reading habits of Turkish high school students and an examination of the efforts to encourage them to read [Doctoral dissertation].* Loughborough: Loughborough University.

[B65] Kuterbach J. M. (2012). *A model of academic enablers and academic performance among postsecondary learners* [Unpublished doctoral dissertation]. Pennsylvania: Penn State University.

[B66] Lawrence A. S. (2014). Relationship between study habits and academic achievement of higher secondary school students. *Online Submission* 4 143–145. 10.15373/2249555X/June2014/43 33525263

[B67] Loan F. A. Shah R. (2017). Survey of the literature reading habits and preferences of adolescents: A study of a public school in India. *Libr. Inform. Sci. Res. Electron. J.* 27 80–96.

[B68] Madhavi S. Naidu S. Krishnaveni A. Kiran P. (2014). Study skills assessment among medical undergraduates-where they stand. *J. Dent. Med. Sci.* 13 16–19. 10.9790/0853-131031619

[B69] Maiyo J. Siahi E. A. (2015). Study of the relationship between study habits and academic achievement of students: A case of Spicer Higher Secondary School, India. *Int. J. Educ. Admin. Pol. Stud.* 7 134–141. 10.5897/IJEAPS2015.0404

[B70] Malik M. Parveen N. (2016). Study habits and academic achievement: A comparative analysis of the high and low academic achievers. *Bahria J. Prof. Psychol.* 15 46–54.

[B71] Mansor A. N. Rasul M. S. Abd Rauf R. A. Koh B. L. (2013). Developing and sustaining reading habit among teenagers. *Asia Pac. Educ. Res.* 22 357–365. 10.1007/s40299-012-0017-1

[B72] Miesen H. W. J. M. (2003). Predicting and explaining literary reading: An application of the theory of planned behavior. *Poetics* 31 189–212. 10.1016/S0304-422X(03)00030-5

[B73] Mirza Q. Pathan H. Khatoon S. Hassan A. (2021). Digital age and reading habits: Empirical evidence from Pakistani Engineering University. *TESOL Int. J.* 16 210–231.

[B74] Mishra P. Pandey C. M. Singh U. Gupta A. Sahu C. Keshri A. (2019). Descriptive statistics and normality tests for statistical data. *Ann. Card. Anaesth.* 22 67–72. 10.4103/aca.ACA_157_18 30648682 PMC6350423

[B75] Molotja T. W. Themane M. (2018). Enhancing learners’ reading habits through reading bags at secondary schools. *Read. Writ.* 9 1–9. 10.4102/rw.v9i1.185

[B76] Myers M. D. (2019). *Qualitative research in business and management.* Thousand Oaks, CA: Sage.

[B77] Naqvi S. Chikwa G. Menon U. Al Kharusi D. (2018). Study skills assessment among undergraduate students at a private university college in Oman. *Mediterr. J. Soc. Sci.* 9 139–147. 10.2478/mjss-2018-0034

[B78] Nguyen Thi Thu H. (2022). The effects of reading habits on writing performance: A case study at Van Lang University. *Int. J. TESOL Educ.* 2 105–133. 10.54855/ijte.22247

[B79] Nouhi E. Shakoori A. Nakhei N. (2009). Study habits and skills, and academic achievement of students in Kerman University of medical sciences. *J. Med. Educ.* 12 77–80. 10.22037/jme.v1213,4.1249

[B80] Ogeyik M. C. Akyay E. (2009). Investigating reading habits and preferences of student teachers at foreign language departments. *Int. J. Lang. Soc. Cult.* 28 72–78.

[B81] Owusu-Acheaw M. Larson A. G. (2014). Reading habits among students and its effect on academic performance: A study of students of Koforidua polytechnic. *Libr. Philos. Pract.* 1 1–22.

[B82] Oyewole O. (2017). Impact of poor reading culture among selected secondary school students in Owo local government area of Ondo state, Nigeria. *Dev. Country Stud.* 7 88–101.

[B83] Palani K. K. (2012). Promoting reading habits and creating a literate society. *J. Arts Sci. Commerce* 3 90–94.

[B84] Pehlivan A. Serin O. Serin N. B. (2010). Determining reading interests and habits of candidate teachers (TRNC Sample). *Procedia Soc. Behav. Sci.* 9 869–873. 10.1016/j.sbspro.2010.12.251

[B85] Pillai S. K. (2012). An empirical study on study habits of X standard students in Nagarkovil district. *Res. Expo Int. Multidiscip. Res. J.* 2 15–27.

[B86] Pintrich P. R. Smith D. A. Garcia T. McKeachie W. J. (1993). Reliability and predictive validity of the Motivated Strategies for Learning Questionnaire (MSLQ). *Educ. Psychol. Meas.* 53 801–813. 10.1177/0013164493053003024

[B87] Porkaew K. Fongpaiboon A. (2018). Effects of extensive reading on Thai tertiary ‘students’ reading attitudes. *Arab World Eng. J. (AWEJ)* 9 207–219. 10.24093/awej/vol9no1.15

[B88] Pretorius E. J. Klapwijk N. M. (2016). Reading comprehension in South African schools: Are teachers getting it, and getting it right? *Per Linguam* 32 1–20. 10.5785/32-1-627

[B89] Quadir B. Chen N. S. (2015). The effects of reading and writing habits on learning performance in a blog learning environment. *Asia Pac. Educ. Res.* 24 635–644. 10.1007/s40299-014-0210-5

[B90] Rasheed S. (2012). ’ Children’s reading habits: A study of Lahore city. *Pak. J. Inform. Manag. Libr.* 5 412–437.

[B91] Rosli N. A. Razali N. F. Zamil Z. U. A. Noor S. N. F. M. Baharuddin M. F. (2018). The determination of reading habits among students: A concept. *Int. J. Acad. Res. Bus. Soc. Sci.* 7 791–798. 10.6007/IJARBSS/v7-i12/3710

[B92] Rozalski M. E. (2008). Practice, practice, practice: How to improve students’ study skills. *Beyond Behav.* 17 17–23.

[B93] Sabbah S. (2016). The effect of study habits on English language achievement. *Arab World Eng. J. (AWEJ)* 7 238–257.

[B94] Schober P. Boer C. Schwarte L. A. (2018). Correlation coefficients: appropriate use and interpretation. *Anesth. Analg.* 126 1763–1768. 10.1213/ANE.0000000000002864 29481436

[B95] Schwabe F. McElvany N. Trendtel M. (2015). The school age gender gap in reading achievement: Examining the influences of item format and intrinsic reading motivation. *Read. Res. Q.* 50 219–232. 10.1002/rrq.92

[B96] Shahidi F. Dowlatkhah H. R. Avand A. Musavi S. R. Mohammadi E. (2014). A study on the quality of study skills of newly-admitted students of Fasa University of Medical Sciences. *J. Adv. Med. Educ. Prof.* 2 45–50. 25512918 PMC4235533

[B97] Sherafat R. Murthy C. V. (2016). A study of study habits and academic achievement among secondary and senior secondary school students of Mysore city. *Int. J. Indian Psychol.* 3 161–170. 10.25215/0302.055

[B98] Silverrajoo P. Hassan A. (2018). Relationship between study habits and academic achievement among health science students. *Int. J. Acad. Res. Bus. Soc. Sci.* 8 763–780. 10.6007/IJARBSS/v8-i7/4418

[B99] Singh Y. G. (2011). Academic achievement and study habits of higher secondary students. *Int. Ref. Res. J.* 3 27–42.

[B100] Stokmans M. J. W. (1999). Reading attitude and its effect on leisure time reading. *Poetics* 26 245–261. 10.1016/S0304-422X(99)00005-4

[B101] Strunk T. A. (2014). *An exploration of the relationships between academic enablers and middle school achievement* [Unpublished doctoral dissertation]. Pennsylvania: Penn State University.

[B102] Tahamtani T. Jalil K. Hosseini M. SoltaniArabshahi K. (2017). Correlation of study habits with academic achievement among students attending the national medical science Olympiad. *J. Adv. Med. Educ.* 3 19–23.

[B103] Thamarasseri I. (2018). Cognitive styles, study habits and academic achievement of students of Central University of Kashmir. *Stud. Home and Commun. Sci.* 12 9–20. 10.31901/24566780.2018/12.1-2.335

[B104] Tonka H. Bakir S. (2020). The examination of the relationship between the secondary school students’ habit of reading and their reading anxiety. *J. Educ. Issues* 6 293–313. 10.5296/jei.v6i1.16986

[B105] Van Schooten E. De Glopper K. Stoel R. D. (2004). Development of attitude toward reading adolescent literature and literary reading behavior. *Poetics* 32 343–386. 10.1016/j.poetic.2004.07.001

[B106] Walia P. K. Sinha N. (2014). Changing trend in reading habits of teenagers in Delhi: An impact assessment of demographic and environmental variables. *Libr Rev.* 63 125–137. 10.1108/LR-03-2013-0038

[B107] Wernersbach B. M. Crowley S. L. Bates S. C. Rosenthal C. (2014). Study skills course impact on academic self-efficacy. *J. Dev. Educ.* 37 14–33.

[B108] Whitten C. Labby S. Sullivan S. L. (2016). The impact of pleasure reading on academic success. *J. Multidiscip. Grad. Res.* 2 48–64.

[B109] Winne P. H. (2013). “Learning strategies, study skills, and self-regulated learning in postsecondary education,” in *Higher education: Handbook of theory and research*, ed. Paulsen M. (Berlin: Springer), 377–403. 10.1007/978-94-007-5836-0_8

[B110] Wu L. Valcke M. Van Keer H. (2019). Factors associated with reading comprehension of secondary school students. *Educ. Sci. Theory Pract.* 19 34–47. 10.12738/estp.2019.4.003

